# CLASRP oncogene as a novel target for colorectal cancer

**DOI:** 10.1007/s10142-023-01208-8

**Published:** 2023-09-02

**Authors:** Quan Gu, Jianzhong Wu, Heng Xu, Haixia Cao, Junying Zhang, Changwen Jing, Zhuo Wang, Mengjie Du, Rong Ma, Jifeng Feng

**Affiliations:** 1 Nanjing Medical University Affiliated Cancer Hospital and Research Center for Clinical Oncology, Jiangsu Cancer Hospital and Jiangsu Institute of Cancer Research, 42 BaiZiTing Road, Nanjing, Jiangsu 210000 People’s Republic of China; 2 Jiangsu Provincial Institute of Materia Medica, Nanjing Tech University, Nanjing, Jiangsu China

**Keywords:** CLASRP, Promotional oncogene, Clk inhibitors, Apoptosis, Colorectal cancer

## Abstract

Clk4-associated serine/arginine-rich protein (*CLASRP*), an alternative splicing regulator, may be involved in the development and progression of cancer by regulating the activity of the CDC-like kinase (Clk) family. This study explored the biological function of *CLASRP* in colorectal cancer (CRC). The expression of *CLASRP*, which is associated with clinicopathological features, was analysed in CRC tissues and paired noncancer tissues by RT–PCR. The roles of *CLASRP* were investigated in CRC cells transfected with plasmids or shRNA through proliferation, migration and invasion assays *in vitro* and a xenograft model *in vivo*. Apoptosis was analysed using *CLASRP*-overexpressing CRC cells by western blotting. Clk inhibitors were used to perform functional research on *CLASRP* in *CLASRP*-overexpressing CRC cells. *CLASRP* was significantly upregulated in CRC cell lines, while high *CLASRP* expression was correlated with metastasis in CRC patients. Functionally, overexpression of *CLASRP* significantly promoted the proliferation, migration and invasion of CRC cells in vitro and tumour growth in vivo. Mechanistically, the proliferation, migration and invasion of *CLASRP*-overexpressing CRC cells were inhibited by Clk inhibitors, accompanied by low expression of CLASRP at the gene and protein levels. Clk inhibitors induced apoptosis of *CLASRP*-overexpressing CRC cells, resulting in direct blockade of cell growth. The expression levels of cleaved caspase 3 and cleaved caspase 8 were increased in *CLASRP*-overexpressing CRC cells treated with Clk inhibitors. *CLASRP* might serve as a promotional oncogene in CRC cells and be suppressed by Clk inhibitors through activation of caspase pathways.

**Supplementary information:**

The online version contains supplementary material available at 10.1007/s10142-023-01208-8.

## Introduction

Colorectal cancer (CRC) is the second most prevalent cause of cancer death and ranks second in terms of the most common cancers diagnosed in men and women in the USA (Siegel et al. 2023a, 2023b). From 2000 to 2018, with the rapid dissemination of colonoscopy screening, colonoscopy prevalence tripled from 20 to 61% among adults over 50 years old (Siegel et al. 2023a, 2023b). In developing countries, improvements in nutrition and lifestyle changes have resulted in increased CRC incidences, similar to that in developed countries 20 years ago (Li and Lai 2009). Although the survival rate of CRC patients has improved through multimodal treatment over the past 30 years, over 50% of patients with CRC further develop metastases, resulting in poor prognosis with a five-year survival rate of 12.5% (Van Cutsem et al. 2014). Therefore, tumour progression must be closely monitored, and new treatment strategies must be explored to improve the prognosis of patients with CRC.

Differences in the gene expression of splicing regulators have been examined in multiple cancers, and their coding proteins often affect the splicing patterns of many other genes that participate in certain cancer-specific biological pathways (El Marabti and Younis 2018; Shkreta et al. 2013). Clk4-associating serine/arginine-rich protein (CLASRP) is a binding partner of Clk4; evidence indicates that it may serve as an AS regulator of the activity of the CDC-like kinase (Clk) family (Katsu et al. 2002). As a candidate gene, CLASRP is an independent prognostic factor for patients with head and neck cancer, and it has been shown to have remarkable functions in cancer development (Liang et al. 2019). Furthermore, CLASRP gene was found to be commonly up-regulated and this was correlated to a poor prognosis of clear cell renal cell carcinoma (Yang et al. 2021). Indeed, CLASRP is a prognostic predictor for head and neck cancer and clear cell renal cell carcinoma. Therefore, CLASRP maybe participates in the progression of cancers; however, very few reports have revealed the gene functions and significance of CLASRP.

In this study, we confirmed CLASRP as a promotional oncogene in the progression of CRC. Functional studies revealed that CLASRP promotes the proliferation, migration and invasion of CLASRP-overexpressing CRC cells in vitro. The role of CLASRP in cell growth, the cell cycle and apoptosis during tumour progression was clarified through treatment with specific Clk inhibitors.

## Materials and methods

### Patient samples

CRC tissues and paired adjacent tissues were collected from 83 patients who underwent tumour resection at Jiangsu Cancer Hospital (Nanjing, China) from October 2019 to December 2020. The tissues were frozen in liquid nitrogen immediately and then stored in a liquid nitrogen tank. CRC tissues and paired adjacent noncancer tissues were identified by histopathology. All patients were enrolled in a research protocol of the Research Center for Clinical Oncology. This protocol was approved by the Biomedical Research Ethics Committee of Jiangsu Cancer Hospital (2018-024). All participants signed informed consent forms.

### Cell lines

The human normal colon epithelial cell line (HCoEpiC) was purchased from Guandao Biology (Shanghai, China). The human CRC cell lines DLD-1, SW480, HCT116 and LoVo were obtained from ATCC (MD, USA). All cells were grown in DMEM (Gibco, MA, USA) with 10% foetal bovine serum (BI, USA) at 37 °C under 5% CO_2_.

### RNA extraction and qRT–PCR

Total RNAs were extracted from CRC cells and tissues via TRIzol reagent (Invitrogen, CA, USA). Complementary DNAs were synthesized using PrimeScript RT Master Mix (Takara, Japan) for RT–PCR. The number of mRNAs was quantified by real-time PCR analyses, which were performed using PowerUp SYBR Green Master Mix (Thermo Fisher Scientific, MA, USA) and a Biosystems 7500 Sequence Detection System (Applied Biosystems). The reactions were carried out under cycling conditions of pre-denaturation at 95°C for 300s, 40 cycles of denaturation at 95°C for 20 s, annealing at 55°C for 20 s and extension at 72°C for 20 s. A comparative Ct method was used to compare each condition to the control reactions. GAPDH was used as an internal control for mRNA RT–PCR and its reliability was determined by the computation tool RefFinder (Xie et al. 2023). The primer sequences are listed in Table S2.

### Construction of stably transfected CRC cell lines

The shRNA sequence with a specific interference effect (sh-CLASRP) was purchased from Ribobio (Guangzhou, China), whereas a scrambled shRNA was used as a negative control. The shRNA sequence is listed in Table S3.

DLD-1 and SW480 cells were infected with GFP and puromycin-resistant lentiviruses that knocked down or overexpressed CLASRP. Lentiviruses and their corresponding controls were purchased from Corues Biotechnology (Jiangsu, China). Stable CRC cell lines with silenced or increased expression of CLASRP were established by puromycin screening. In addition, the gene knockout cell line (KO) used in this study was constructed by Corues Biotechnology (Jiangsu, China) in DLD-1 cells using CRISPR–Cas9 genome engineering technology and identified by sequencing. Western blot and qRT–PCR analyses were used to identify the efficiency of the stable CRC cell line.

### Cell counting kit-8 (CCK-8) assays

The transfected cells were seeded into 96-well plates at 3 × 10^3^ cells/well. CCK-8 reagent (Dojindo Molecular Technologies, Inc., Kumamoto, Japan) was added to the test (10 μL/well) and incubated for 1 h at 0, 24, 48 and 72 h after transfection. Absorbance was measured at a wavelength of 450 nm (Thermo Fisher Scientific, MA, USA). The effects of inhibitors on the viability and proliferation of CLASRP-overexpressing CRC cells and control vector cells were also assessed. In brief, inhibitor (inhibitor TG003 and inhibitor KH-CB19; APExBIO, USA) preparations were serially diluted with DMEM to achieve final concentrations of 25, 50, 100 and 200 μM. Growth inhibition curves were plotted as percentages of untreated control cells relative to the standard curves, and half-maximal inhibitory concentrations (IC_50_) were calculated.

### Western blotting

Western blot analyses were conducted in accordance with standard protocols. A 200-μL volume of lysis buffer (RIPA: PMSF = 100:1) was added to each well of a 6-well plate and lysed on ice for 30 min. After centrifugation at 12,000 rpm at 4°C for 15 min, the protein concentration was measured using the BCA method. Anti-CLASRP antibody was purchased from Novus Biologicals (USA). These membranes were blocked with 5% bovine serum albumin for 1 h and then probed with specific anti-CDK1 (1:500; mouse; cat. no. ab18; Abcam, Cambridge, England), anti-cleaved caspase-3 (1:1000; rabbit; cat. no. 9661; Cell Signaling Technology, MA, USA), anti-caspase-8 (1:1000; rabbit; cat. no. 4790; Cell Signaling Technology, MA, USA), anti-cleaved caspase-8 (1:1000; rabbit; cat. no. 9496; Cell Signaling Technology, MA, USA) and anti-Bcl-XL (1:1000; rabbit; cat. no. 2764; Cell Signaling Technology, MA, USA). The protein levels were standardized by detecting the same blots with β-actin antibody (Santa Cruz Biotechnology, CA, USA).

### Tumour xenograft

Six-week-old BALB/c nude mice with body weights of 19–22 g (female) were purchased from GemPharmatech Co., Ltd. (Nanjing, China) and randomly divided into two groups (*n* = 8). All protocols were approved by the Institutional Animal Care and Use Committee at Nanjing Medical University. CLASRP-overexpressing DLD-1 cells (2.5 × 10^6^ cells, CLASRP overexpression group) or CLASRP knockout DLD-1 cells (5 × 10^6^ cells, CLASRP knockout group) and corresponding control cells (control groups) in 200 μL of PBS with Matrigel (Corning, USA) were injected subcutaneously into the leg flank of nude mice. Tumour volume was measured every 3 days postinjection and calculated as 0.5 × length × (width)^2^. After slaughter, tumours were weighed and collected for total RNA extraction, and the remainder was fixed in 4% paraformaldehyde for 24 h and then processed for haematoxylin and eosin staining and immunohistochemical (IHC) staining for CLASRP. In addition, after the xenograft models were established, one group was treated with the inhibitor TG003 (TG003 treatment groups). Isotype solvent was used as a control (control group). The inhibitor TG003 (i.p., 25 mg/kg) was administered once every 3 days.

### Cell migration and invasion assays in vitro

The transfected cells (2×10^5^) were spread in the upper chambers of Transwell assay inserts (Corning, USA) containing serum-free DMEM, and the migration was tested by membrane. The lower chambers were filled with DMEM containing 20% FBS. The cells on the filter surface were fixed with 4% paraformaldehyde and stained with crystal violet. After 24 h, they were photographed with a digital microscope. The transfected cells (2×10^5^) were also spread in the top chamber containing a Matrigel-coated membrane (Corning, USA) in serum-free DMEM to detect cell invasion. The bottom chambers were also filled with 20% FBS-DMEM. The invasion ability was measured after 48 h. The number of migrated and invaded cells was counted in three randomly selected fields.

### Wound healing assays

DLD-1 and SW480 cells were seeded into 6-well plates at 5 ×10^5^ cells/well. Overexpression plasmids or oligonucleotides were transfected when cell confluency reached 80%. Wounds were made with the fine end of a 10-μL pipette tip at 24 h after transfection. The area occupied by the migratory cells was estimated according to photographs taken at the appropriate time.

### Cell cycle analysis and cell apoptosis analysis

CLASRP-overexpressing CRC cells were cultured in different concentrations of inhibitors (25, 50 and 100 μM) for 72 h at 37 °C under 5% CO_2_. Inhibitor-free and CLASRP-overexpressing CRC cells were used as controls. After harvesting, the cells were fixed with 70% ethanol at −20 °C overnight and then stained with 50 μg/mL propidium iodide (PI) (KeyGEN BioTECH, China) containing 0.25 mg/mL RNase A (KeyGEN BioTECH, China) for 30 min at room temperature. In addition, the cells were washed twice with ice-cold PBS and incubated in 5 μL of annexin V-APC (KeyGEN BioTECH, China) and 5 μL of PI for 15 min at room temperature in the dark. Analysis was performed using a FACS-CaliburTM instrument equipped with CELLQuestTM software (Becton Dickinson, NJ, USA).

### Pathology and histochemistry

Pathological changes in patient tissues and tumour xenografts excised from nude mice were evaluated by a professional clinician. Samples of these tissues were fixed in 10% neutral buffered formalin for 24 h, dehydrated, embedded in paraffin wax and sectioned (3−5 μm). The sections were mounted on conventional glass slides for histochemistry studies. All sections were stained with HE (KeyGEN BioTECH, China). IHC detection was performed on tumour xenografts using rabbit anti-CLASRP antibody (Novus, USA), and staining was performed with a MaxVision kit (MXB Biotechnologies, China). Immunofluorescence assay (IFA) was performed on patient tissues using FITC-labelled goat anti-rabbit antibody as the secondary antibody (Jackon ImmunoResearch, USA) after incubation with rabbit anti-CLASRP antibody (Novus, USA). Finally, the sections were dehydrated and sealed, and images were captured with an Olympus FSX100 microscope (Olympus, Japan).

### Statistical analysis

Each experiment had at least three biological replicates or technical replicates. Numerical data are presented as the mean ± SD with three determinations. The comparisons between two groups of normalized data were analysed by *t* tests. Comparisons for ≥ 3 groups were conducted using ANOVA, followed by pairwise comparisons using Bonferroni post hoc tests. *P* < 0.05 was considered statistically significant with 95% confidence.

## Results

### CLASRP is overexpressed in CRC tissues from patients with metastasis

**Fig. 1:**
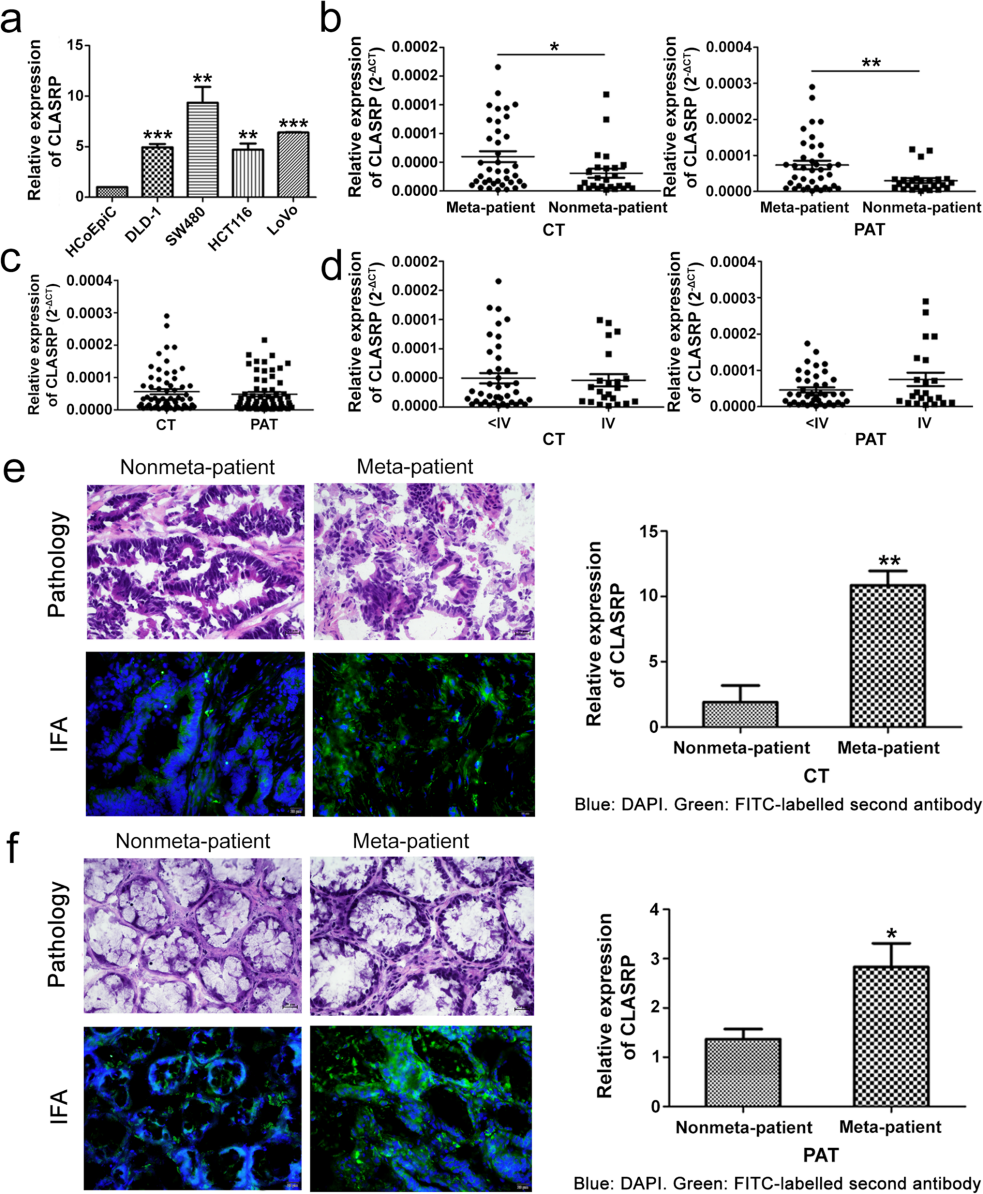
Analysis of CLASRP expression in CRC cells and tissues. **a** CLASRP expression in CRC cells. **b** CLASRP expression in CRC tissues and paired adjacent tissues. CLASRP expression in CRC tissues and paired adjacent tissues from patients with **c** metastasis and non-metastasis and **d** IV and <IV stage. The expression of CLASRP in **e** CRC tissues and **f** paired adjacent tissues from non-metastatic and metastatic patients by IFA. Blue: DAPI. Green: FITC. CT, CRC tissues. PAT, paired adjacent tissues. Meta-patient, patients with metastasis. Nonmeta-patient, patients with non-metastasis. Scale bar, 20 μm. Data are mean ± SD, *n* = 3. ***P < 0.001, **P < 0.01, *P < 0.05

Compared to the normal colon epithelial cell line HCoEpiC, CLASRP was significantly overexpressed in CRC cells, such as DLD-1, sw480, HCT116 and LoVo (*P* < 0.01; Fig. 1a). To investigate the expression levels of CLASRP in tissues, CRC tissues and paired adjacent tissues from 83 CRC patients were randomly selected. The expression of CLASRP in CRC tissues from patients with non-metastasis was lower than those in patients with metastasis (*P* < 0.05; Fig. 1b). Meanwhile, patients with metastasis had significantly high CLASRP expression in paired adjacent tissues compared to patients with non-metastasis (*P* < 0.01; Fig. 1b). However, no significant difference was found in the expression of CLASRP between CRC tissues and paired adjacent tissues by RT-PCR assay (*P* > 0.05; Fig. 1c). No significant differences were found in the expression of CLASRP between stage IV and stage <IV patients in CRC tissues and paired adjacent tissues (*P* > 0.05; Fig. 1d). Since EMT (epithelial to mesenchymal transition) is closely related to CRC metastasis (Wei et al. 2019), and EMT markers might indicate early occurrence of cancer metastasis (Zhang et al. 2021), it is also addressed that EMT-related associated genes might predict the prognosis and the response to immunotherapy (Gao et al. 2022). We also checked the EMT markers in tumour tissues from non-metastatic and metastatic patients by RT-PCR assay. The expressions of N-cadherin and Vimentin were significantly lower in nonmetastatic patients than those in metastatic patients. However, metastatic patients had a great lower expression of E-cadherin (*P*<0.05; Fig. S1). These results confirmed that the expression of CLASRP was upregulated in tumour tissues from metastatic patients.

CLASRP has been shown to be relevant to the poor prognosis of patients with clear cell renal cell carcinoma (Yang et al. 2021) and to be an independent prognostic factor for patients with head and neck cancer. However, the correlation between clinical value and CLASRP in patients with CRC has not been investigated. In this study, clinicopathological analysis of 83 patients with CRC revealed that the expression of CLASRP was correlated with metastasis, including lymph node and distant metastasis, and CEA concentrations (*P* < 0.05) but not with age, gender or TNM stage (*P* > 0.05; Table 1).

**Table 1:** Correlation between expression of CLASRP and the clinicopathological features of patients with colorectal cancer

Variables	Cases(*n*)	*CLASRP*		*P* value
	(Total *n*=83)	Low (*n*)	High (*n*)	
Age (years)				
≤60	42	20	22	0.222
>60	41	25	16	
Gender				
Male	55	31	24	0.582
Female	28	14	14	
TNM stage				
<IV	57	28	29	0.168
IV	26	17	9	
Metastasis				
Yes	47	16	31	0.014
No	36	14	22	
Differentiation				
High	1	1	0	0.355
Middle–low	82	44	38	
CEA (μg/L)				
<5	45	18	27	0.005
≥5	38	27	11	

Low and high, low *CLASRP*-expression, and high *CLASRP*-expression (CRC tissues vs paired adjacent tissues)

Furthermore, histochemistry verified the expression of CLASRP in CRC tissues and paired adjacent tissues from non-metastatic patients and metastatic patients. Green fluorescence represents the expression quantity of CLASRP in CRC tissues and paired adjacent tissues. IFA revealed that the expression levels of CLASRP in CRC tissues from patients with metastasis were evidently stronger than those in CRC tissues from patients without metastasis. Similarly, the expression levels of CLASRP in paired adjacent tissues from patients with metastasis were also high compared to those from patients without metastasis (*P* < 0.05; Fig. 1e, f). These results revealed that CLASRP might play an important role in the progression of CRC, such as in metastasis.

### CLASRP promotes CRC cell migration and proliferation in vitro

**Fig. 2:**
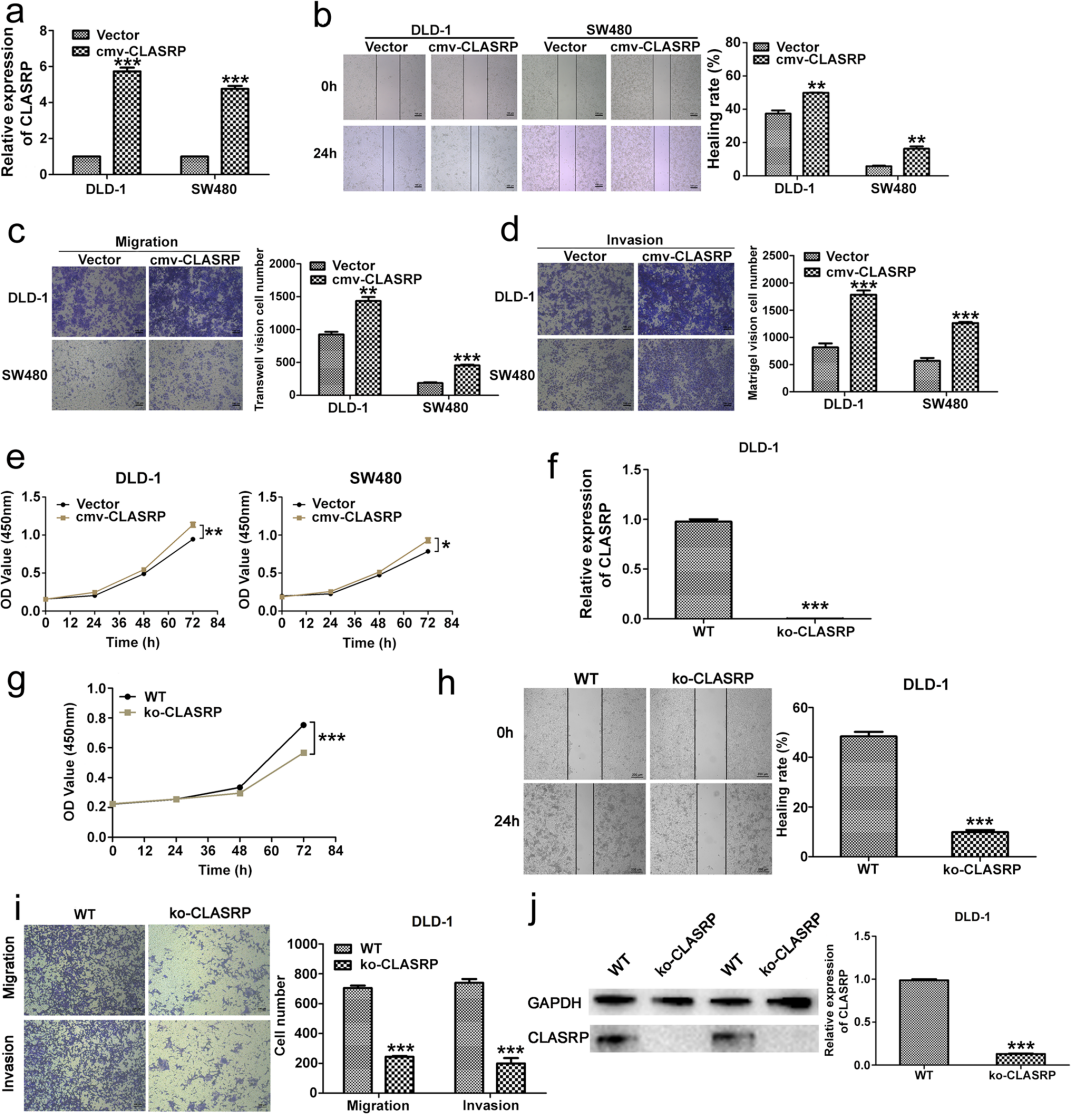
The phenotypic and functional characteristics in CLASRP overexpression and knockout CRC cells. **a** CLASRP expression was measured by qRT-PCR in DLD-1 and SW480 cells after transfected overexpression plasmid. **b**–**e** The biological role of CLASRP on cell migration, invasion and proliferation capability was assessed by wound healing, Transwell migration and Matrigel invasion assay and CCK-8 assays in CLASRP-overexpressed DLD-1 and SW480 cells. **f** CLASRP expression was measured by qRT-PCR in CLASRP knockout DLD-1cells. **g**–**i** The biological role of CLASRP on proliferation capability, cell migration and invasion was assessed by CCK-8 assays, wound healing, Transwell migration and Matrigel invasion assay in CLASRP knockout DLD-1 cells. **j** The protein expression of CLASRP in CLASRP knockout DLD-1 cells. WT, wild type as a control. Data are mean ± SD, *n* = 3. ***P < 0.001, **P < 0.01, *P < 0.05

After transfection of the CLASRP-overexpression lentivirus, the expression of CLASRP was notably upregulated in DLD-1 and SW480 cells (*P* < 0.001; Fig. 2a). Wound healing assays showed elevated healing rates after CLASRP overexpression in DLD-1 and SW480 cells (*P* < 0.01; Fig. 2b). Transwell and Matrigel assays illustrated that CLASRP overexpression significantly increased cell migration and invasion in DLD-1 and SW480 cells (*P* < 0.01; Fig. 2c, d). Furthermore, the proliferation of DLD-1 and SW480 cells was promoted after CLASRP overexpression in the CCK-8 assay (*P* < 0.01; Fig. 2e).

Subsequently, we designed shRNAs targeting CLASRP to determine their function. The expression of CLASRP was significantly downregulated in DLD-1 and SW480 cells after transfection of the shRNA (*P* < 0.01; Fig. S2a). Functional phenotype assays showed that the migratory, invasive and proliferative capabilities of CRC cells were suppressed after CLASRP knockdown (*P* < 0.01; Fig. S2b–e).

Further studies were conducted with CLASRP knockout DLD-1 cell lines. The expression of CLASRP was significantly downregulated after CLASRP knockout (*P* < 0.001; Fig. 2f). Proliferation was prominently inhibited at 72 h (*P* < 0.001; Fig. 2g). Similarly, the migratory and invasive capabilities were significantly restrained (*P* < 0.001; Fig. 2h, i). The protein expression of CLASRP was also downregulated (*P* < 0.001; Fig. 2j).

### CLASRP promotes the growth of CRC cells in vivo

**Fig. 3:**
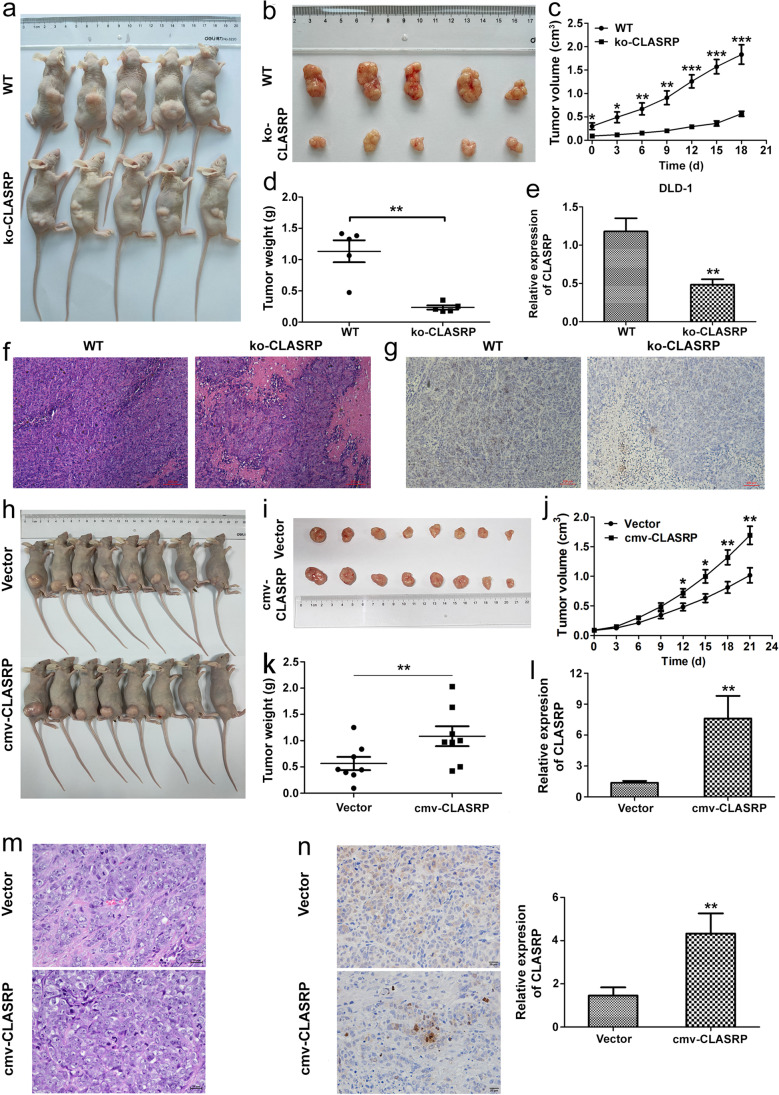
Overexpression of CLASRP promoted the growth of CRC cells in vivo. **a**, **b** Photographs of tumours from CLASRP knockout DLD-1 cells inoculate group and the control mice (*n* = 5). **c** The growth rate and **d** tumour weight decreased in CLASRP knockout group compared with the control group. **e** CLASRP expression decreased in xenografted tumours of CLASRP knockout group compared with the control group. The differences in **f** HE staining and **g** IHC examination. **h**, **i** DLD-1 cells transfected with CLASRP plasmids or empty vectors were subcutaneously injected into the right legs of nude mice (*n* = 8). **j** Tumour weight and **k** the growth rate increased in CLASRP overexpression group compared with the control group. **l** CLASRP expression increased in xenografted tumours of overexpression group compared with the control group. **m** HE staining and **n** IHC examination showed that high expression of CLASRP in CLASRP overexpression group. Data are mean ± SD. *n* =3. **P < 0.01, *P < 0.05

Compared to the control group, the KO-CLASRP group exhibited a significant reduction in tumour volume (*P* < 0.001; Fig. 3a–c). Moreover, tumour weight was reduced in the KO-CLASRP group (*P* < 0.01; Fig. 3d). The expression of CLASRP was significantly downregulated in the KO-CLASRP group compared with the control group (*P* < 0.01; Fig. 3e). Histological analysis revealed differences between the KO-CLASRP and control groups, including low expression of CLASRP in the KO-CLASRP group by IHC (Fig. 3f, g). The results showed that the growth of DLD-1 xenografts was inhibited after CLASRP knockout.

However, the growth of DLD-1 xenografts after CLASRP overexpression was considerably faster than that in the control group (*P* < 0.01; Fig. 3h–j). The tumour weight in the cmv-CLASRP group was notably heavier than that in the control group (*P* < 0.05; Fig. 3k). The expression of CLASRP in tumour tissues of the cmv-CLASRP group was notably higher than that in tumour tissues of the control group (*P* < 0.05; Fig. 3l). HE staining revealed that the tumour scores of the cmv-CLASRP group were higher than those of the control group (Fig. 3m and Table S1). IHC results demonstrated that the expression level of CLASRP in the cmv-CLASRP group was higher than that in the control group (*P* < 0.05; Fig. 3n). These data showed that the growth of DLD-1 cells was enhanced after CLASRP overexpression, corroborating that CLASRP acted as a promotional oncogene.

### Clk inhibitors significantly inhibit the growth of CLASRP-overexpressing CRC cells

**Fig. 4:**
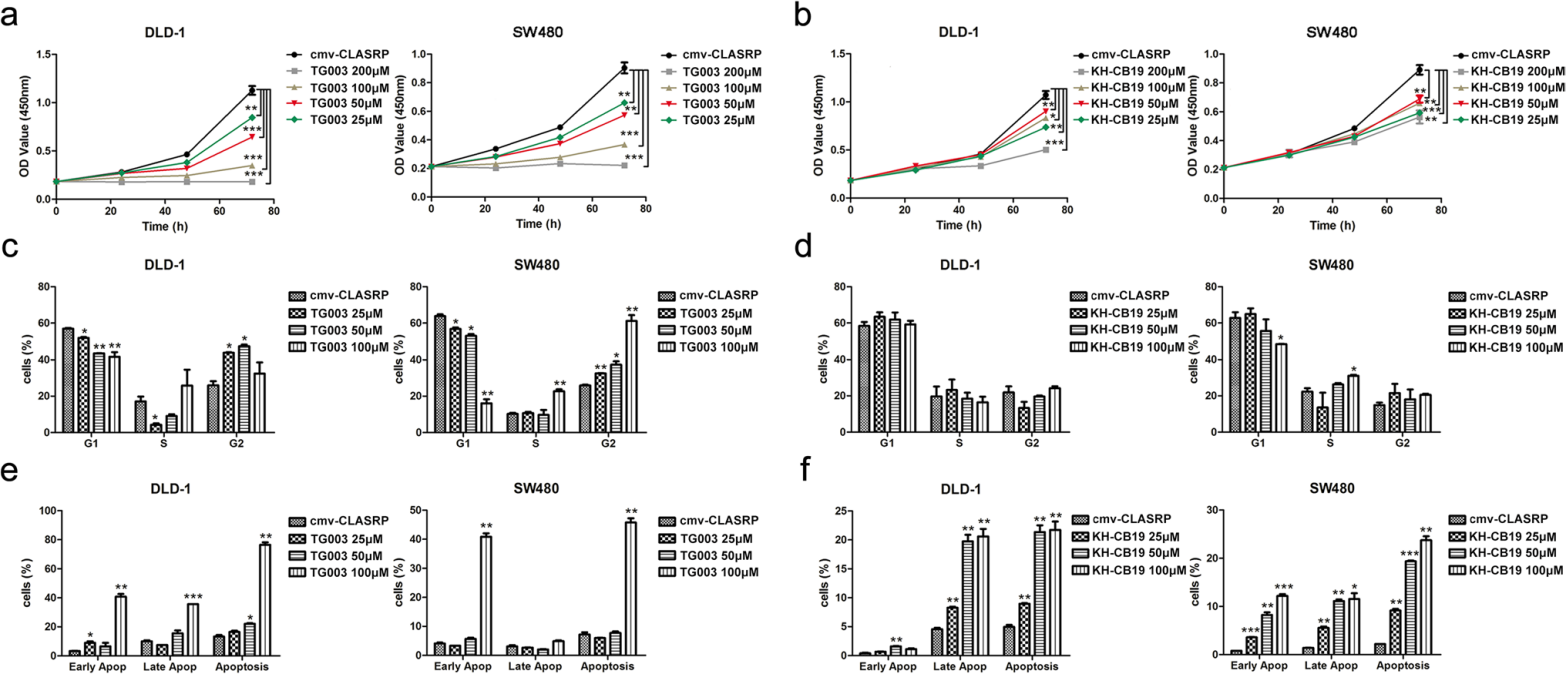
The effects of Clk inhibitors on growth in CLASRP-overexpressed CRC cells. CCK-8 assays in CLASRP-overexpressed CRC cells treated with **a** inhibitor TG003 and **b** inhibitor KH-CB19. The percentage of cell cycle population in CLASRP-overexpressed CRC cells treated with **c** inhibitor TG003 and **d** inhibitor KH-CB19. Percentage of early apoptotic cells and late apoptotic cells in CLASRP-overexpressed CRC cells treated with **e** inhibitor TG003 and **f** inhibitor KH-CB19. cmv, CLASRP- overexpressed CRC cells without treatment. Data are mean ± SD. *n* =3. ***P < 0.001, **P < 0.01, *P < 0.05

Two kinds of inhibitors, namely, a competitive Clk inhibitor (TG003) and a Clk1/4 selective inhibitor (KH-CB19), were chosen to identify the potential effect of CLASRP on cell growth in CRC cells overexpressing CLASRP. The growth of CLASRP-overexpressing CRC cells was significantly suppressed by the inhibitors TG003 and KH-CB19, and the inhibition ratio increased gradually in a dose-dependent manner (*P* < 0.001; Fig. 4a, b). The IC_50_ values of CLASRP-overexpressing DLD-1 cells to inhibitors were lower than those of the controls, suggesting that DLD-1 cells became more sensitive to Clk inhibitors after CLASRP overexpression (*P* < 0.05; Fig. S3a). The reason for this may be that CLASRP is a target of Clk-associated inhibitors. CLASRP overexpression may promote the growth of CRC cells. However, CLASRP-overexpressing DLD-1 cells were more easily inhibited by Clk-associated inhibitors. CLASRP-overexpressing SW480 cells had slightly lower IC_50_ values for Clk-associated inhibitors than vector cells, but the difference was not significant (*P* > 0.05; Fig. S3b).

The cell cycle of CRC cells with CLASRP overexpression was analysed after treatment with inhibitors (100, 50 and 25 μM) for 24 h. Compared with the control group, CLASRP-overexpressing DLD-1 cells treated with the inhibitor TG003 exhibited significant declines in the G1 phase in a concentration-dependent pattern (*P* < 0.01; Fig. 4c, Fig. S4a) and marked arrest in the G2 phase (*P* < 0.01; Fig. 4c, Fig. S4a). Similar results were observed in CLASRP-overexpressing SW480 cells (*P* < 0.01; Fig. 4c, Fig. S4b). However, the effects of the inhibitor KH-CB19 on cell cycle distribution were weak in CLASRP-overexpressing CRC cells (Fig. 4d, Fig. S4c, d). A mechanistic study regarding the role of CDK1 was also conducted given the importance of cyclin-dependent kinase inhibitor (CDK) in promoting transitions through the cell cycle (Lim and Kaldis 2013; Malumbres 2014). The expression of CDK1 declined in vector cells (*P* < 0.05; Fig S5a) but significantly decreased in CLASRP-overexpressing DLD-1 cells at 48 h after treatment with the inhibitors TG003 and KH-CB19 (*P* < 0.01; Fig. S5b). CDK1 is essential for cell division in an embryo because it mainly regulates the transition from G2 phase to M phase in the cell cycle (Malumbres and Barbacid 2009). Cell cycle arrest and CDK1 regulation play important roles in the apoptosis of tumour cells (Kim et al. 2020; Zhao et al. 2019). The apoptosis of CLASRP-overexpressing CRC cells was induced by Clk inhibitors, which could intervene in CLASRP expression.

Thus, the abilities of the inhibitors TG003 and KH-CB19 to induce apoptosis in CLASRP-overexpressing CRC cells were further investigated by flow cytometry. The inhibitor TG003 induced early and late apoptosis in CLASRP-overexpressing DLD-1 cells, while the total apoptotic ratio was approximately 75% at 100 μM (*P* < 0.01; Fig. 4e, Fig. S6a). Compared with the control group, CLASRP-overexpressing SW480 cells treated with a high concentration of the inhibitor TG003 (100 μM) exhibited a significant increase in early apoptosis (*P* < 0.01; Fig. 4e, Fig. S6b). Furthermore, the ratios of early and late apoptosis significantly increased in CLASRP-overexpressing DLD-1 and SW480 cells treated with the inhibitor KH-CB19 in a concentration-dependent manner (*P* < 0.05; Fig. 4f, Fig. S6c, d). These results indicated that the growth of CLASRP-overexpressing CRC cells was blocked due to apoptosis induced by Clk inhibitors.

### CLASRP expression is suppressed by Clk inhibitors in both vector cells and CLASRP-overexpressing DLD-1 cells

**Fig. 5:**
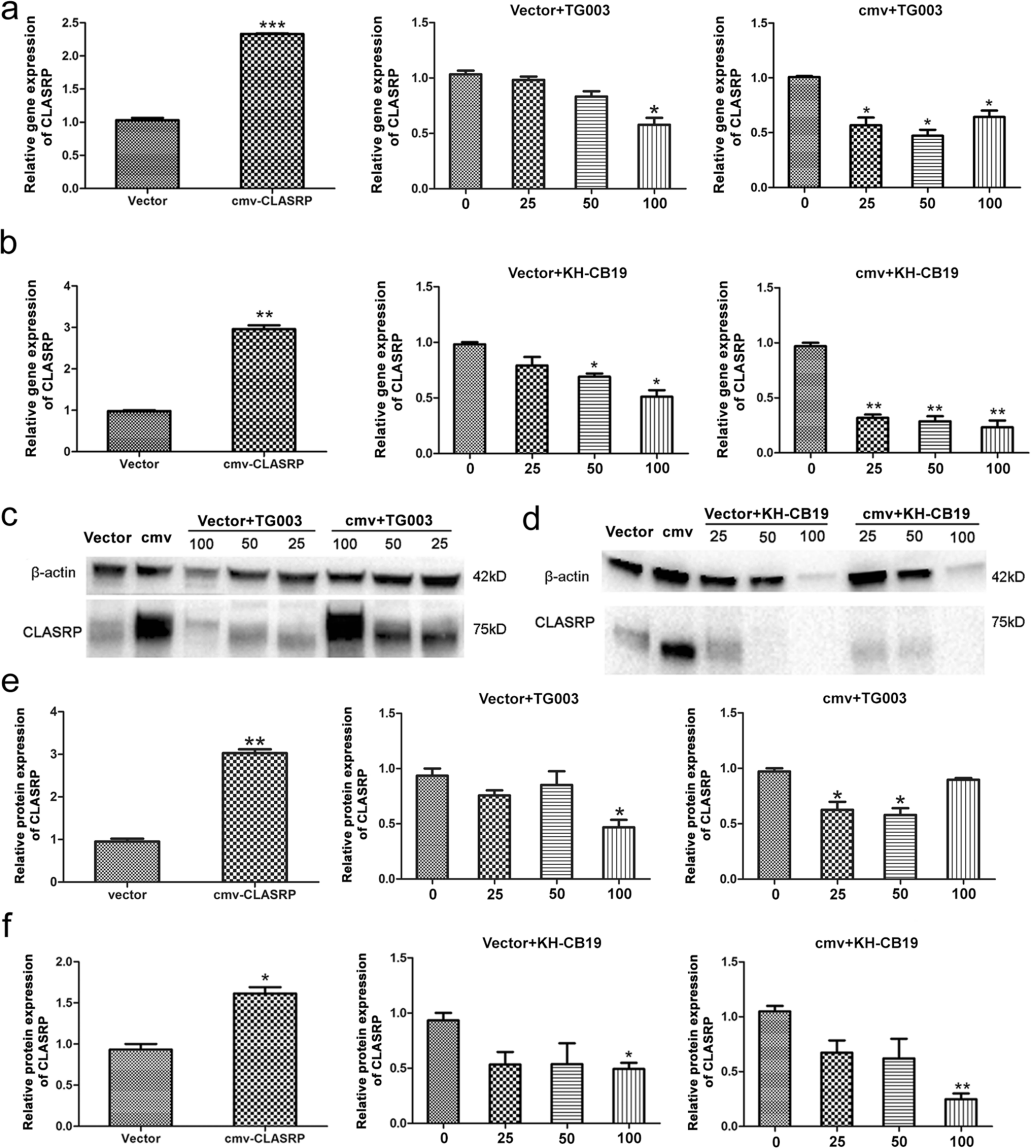
The effects of Clk inhibitors on the expression of CLASRP gene and CLASRP protein in vector and CLASRP-overexpressed DLD-1 cells. The expression of CLASRP gene in vector and CLASRP-overexpressed DLD-1 cells treated with **a** inhibitor TG003 and **b** inhibitor KH-CB19. Immunoblot of vector and CLASRP-overexpressed DLD-1 cells treated with **c** inhibitor TG003 and **d** inhibitor KH-CB19. The protein expression of CLASRP in vector and CLASRP-overexpressed DLD-1 cells treated with **e** inhibitor TG003 and **f** inhibitor KH-CB19. cmv, CLASRP-overexpressed DLD-1 cells without treatment. Data are mean ± SD. *n* =3. **P < 0.01, *P < 0.05

We also investigated the gene and protein expression levels of CLASRP in vector cells and CLASRP-overexpressing DLD-1 cells after treatment with inhibitors. The gene expression of CLASRP decreased at high concentrations of the inhibitor TG003 in vector cells (*P* < 0.05), while significant differences were observed in CLASRP-overexpressing DLD-1 cells (*P* < 0.05; Fig. 5a). The protein expression of CLASRP decreased at 25 and 50 μM TG003 in CLASRP-overexpressing DLD-1 cells (*P* < 0.05), but no difference was observed in vector cells except at 100 μM TG003 (*P* > 0.05; Fig. 5c, e). The inhibitor TG003 could restrain the protein expression of CLASRP in not only CLASRP-overexpressing DLD-1 cells but also vector cells. These findings suggested that CLASRP expression was regulated by the inhibitor TG003 in DLD-1 cells.

In addition, the gene expression levels of CLASRP were suppressed at 50 and 100 μM of the inhibitor KH-CB19 in vector cells (*P* < 0.05; Fig. 5b), whereas the gene expression of CLASRP was significantly downregulated in CLASRP-overexpressing DLD-1 cells treated with the inhibitor KH-CB19 (*P* < 0.01; Fig. 5b). Similarly, the protein expression of CLASRP was significantly decreased in CLASRP-overexpressing DLD-1 cells treated with the inhibitor KH-CB19, especially at 100 μM KH-CB19 (*P* < 0.01; Fig. 5d, f). Overall, these results indicated that the inhibitors TG003 and KH-CB19, targeting the expression of the CLASRP gene or protein, may inhibit the growth of CLASRP-overexpressing CRC cells.

### An abnormal apoptosis signal is induced by Clk inhibitors in both vector cells and CLASRP-overexpressing DLD-1 cells

**Fig. 6:**
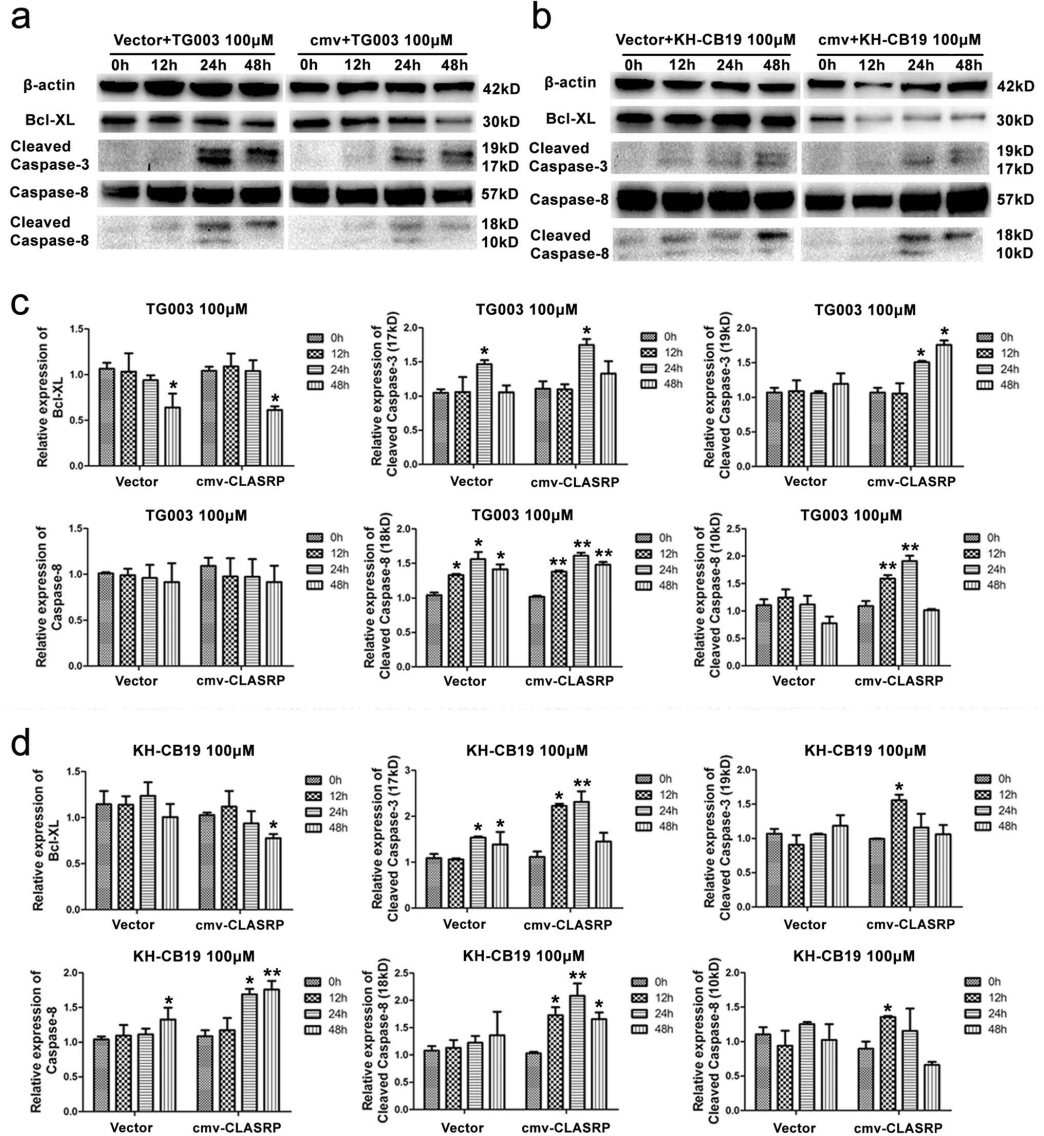
Immunoblot analysis of apoptosis factors in CLASRP-overexpressed DLD-1 treated with Clk inhibitors. Time-dependent effects of **a** inhibitor TG003 and **b** inhibitor KH-CB19 on the expressions of apoptosis factors in vector and CLASRP-overexpressed DLD-1 cells. Analysis of the effect of **c** inhibitor TG003 and **d** inhibitor KH-CB19 on the expression of apoptosis factors in vector and CLASRP-overexpressed DLD-1 cells. cmv, CLASRP-overexpressed DLD-1 cells without treatment. Data are mean ± SD. *n* =3. **P < 0.01, *P < 0.05

The factors regulating apoptosis were identified using lysates of vector control cells and CLASRP-overexpressing DLD-1 cells treated with the inhibitors by Western blot analysis. Compared with the expression levels at 0 h, the expression levels of cleaved caspase-3 (19 KD) increased at 24 h after treatment with the inhibitor TG003 in both vector DLD-1 cells and CLASRP-overexpressing DLD-1 cells (*P* < 0.05; Fig. 6a, c). However, the expression of cleaved caspase-3 (19 KD) gradually increased with prolonged treatment time in CLASRP-overexpressing DLD-1 cells treated with the inhibitor TG003 (*P* < 0.01; Fig. 6a, c). Only the levels of cleaved caspase-8 (18 KD) increased in vector DLD-1 cells (*P* < 0.05), whereas the expression levels of cleaved caspase-8 (10 KD and 18 KD) were significantly activated in CLASRP-overexpressing DLD-1 cells treated with the inhibitor TG003 (*P* < 0.01; Fig. 6a, c). Although apoptotic proteins were also activated in vector DLD-1 cells, the degrees of changes in the expression levels of apoptotic proteins were more intense in CLASRP-overexpressing DLD-1 cells treated with the inhibitor TG003.

Similarly, cleaved caspase-3 (17 kDa), caspase-8 and cleaved caspase-8 (18 kDa) were significantly activated in CLASRP-overexpressing DLD-1 cells treated with the inhibitor KH-CB19 (*P* < 0.01; Fig. 6b, d). Furthermore, the levels of Bcl-XL decreased at 48 h in CLASRP-overexpressing DLD-1 cells treated with the inhibitors TG003 and KH-CB19, suggesting that Clk inhibitors could successfully induce apoptosis in CRC cells overexpressing CLASRP. Caspases play critical roles in apoptosis. Once activated, irreversible biochemical and morphological changes occur (Salvesen and Riedl 2008). The activation of caspase-3 is a central event in the process of apoptosis (Budihardjo et al. 1999; Thornberry and Lazebnik 1998; Wolf and Green 1999). This cysteine protease, which is proteolytically activated by the cleavage of pro-caspase-3 by caspase-8, cleaves several intracellular proteins (Madide et al. 2021; Thornberry et al. 1997). Therefore, the results confirmed that the Clk inhibitor could more severely induce apoptosis of CRC cells overexpressing CLASRP.

### Inhibitor TG003 inhibits the growth of DLD-1 cells by downregulating CLASRP in vivo

**Fig. 7:**
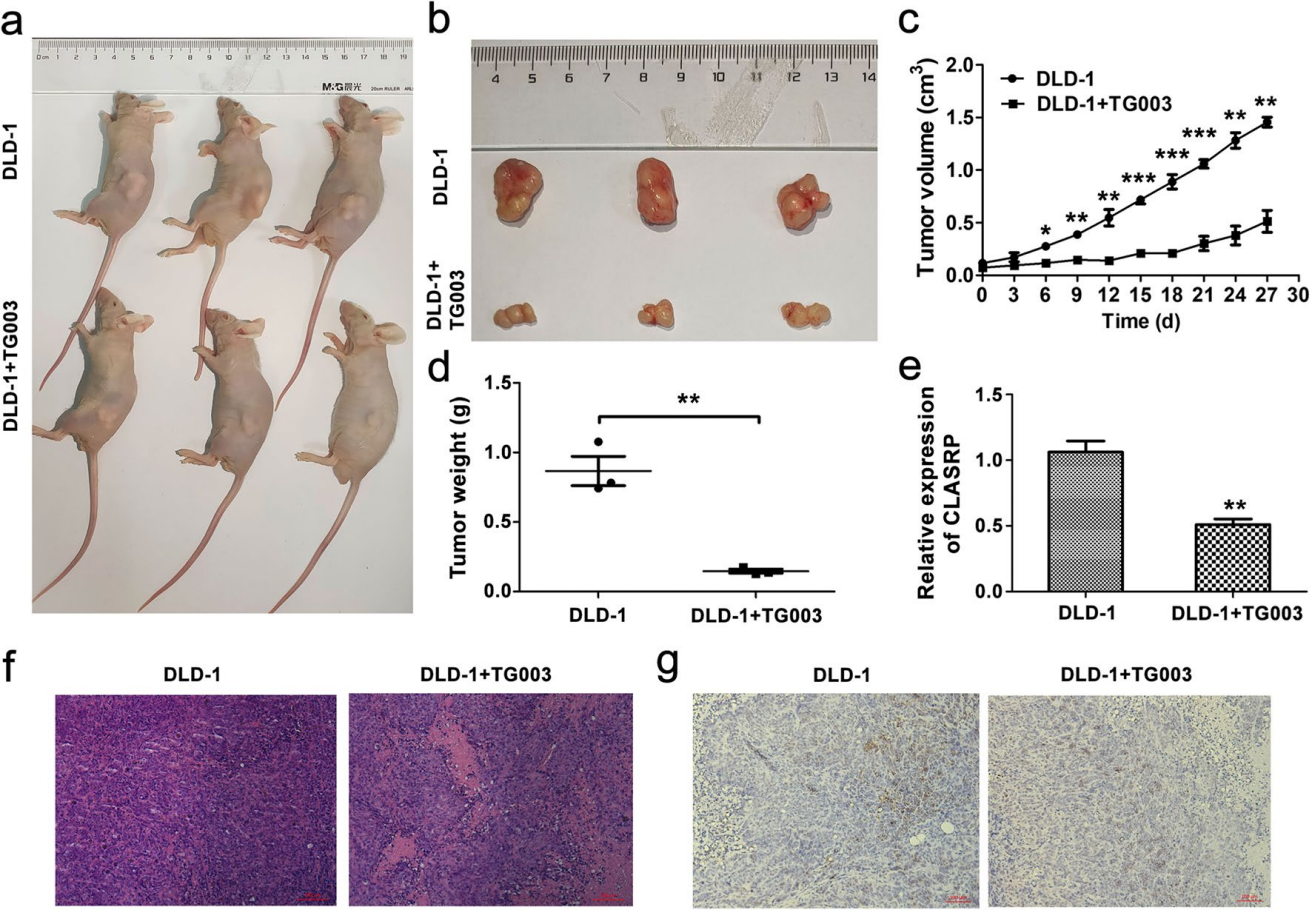
Inhibitor TG003 significantly inhibited the growth of DLD-1 cells in vivo. **a**, **b** Photographs of tumours from inhibitor TG003 treatment group and the control in xenografts with DLD-1 cells (*n* = 3 each group). **c** The growth rate and **d** tumour weight decreased in TG003 treatment group compared with the control group. **e** CLASRP expression decreased after inhibitor TG003 treatment in xenografts with DLD-1 cells. The differences in **f** HE staining and **g** IHC examination. Isotype solvent was used as the control. Data are mean ± SD. *n* =3. **P < 0.01, *P < 0.05

To investigate the correlation between inhibitors and CLASRP, we used the inhibitor TG003 in the in vivo antitumour evaluation of BALB/c nude mice with the DLD-1 tumour xenograft model. The growth of the DLD-1 xenograft was inhibited by the inhibitor TG003 compared with the control (*P* < 0.01; Fig. 7a–c). The tumour weight in the TG003 treatment group was lower than that in the control group (*P* < 0.01; Fig. 7d). The expression of CLASRP was downregulated in the TG003 treatment group compared with the control group (*P* < 0.01; Fig. 7e). Histological analysis showed the differences between the TG003 treatment group and the control, including low expression of CLASRP in the TG003 treatment group by IHC (Fig. 7f, g). The inhibitor TG003 exhibited more potent antitumour activity with significant downregulation of CLASRP, indicating that the inhibitor TG003 significantly inhibited the growth of DLD-1 cells, accompanied by the downregulation of CLASRP expression.

## Discussion

Numerous studies have shown that the disorder of splicing regulation is closely related to the occurrence of diseases, whereas splicing patterns greatly influence cancer progression. As the key recognition factor of pre-mRNA cis-acting elements, serine and arginine-rich splicing factors (SRs) are concentrated in splices and play a vital role in regulating AS (Jeong 2017). CLASRP, as an AS regulator, has rarely been reported in the progression of CRC. Our study indicated that CRC cells overexpressing CLASRP exhibited stronger migration and invasion abilities and grew faster in vitro and in vivo, suggesting that CLASRP may be an effective promotional oncogene that promotes the progression of CRC. These results were consistent with the clinical characteristics, in which the expression of CLASRP was significantly upregulated in CRC tissues and paired adjacent tissues from patients with metastasis.

The amino acid sequences of Clk1 and Clk4 are highly similar. They may act as guardians to maintain the phosphorylation status of SR proteins to improve the survival of cells exposed to stress (Martin Moyano et al. 2020; Ninomiya et al. 2020). The elevated expression of these splicing regulators, such as Clk1 and Clk3, helps cells adapt through the AS of key cancer-associated genes (Bowler et al. 2018). The kinases of the Clk family dominate the supply of full-length, functional mRNA coding for various proteins that are essential for growth and survival in cancer cells (Bu et al. 2020). Thus, the inhibition of Clks may become a novel anticancer strategy, resulting in the selective depletion of cancer-relevant proteins after turnover (ElHady et al. 2017). Clk/Sty kinase directly regulates the activity and compartmentalization of SR splicing factors (Colwill et al. 1996). TG003 inhibits exon skipping, serine/arginine-rich protein phosphorylation and Clk1/Sty-dependent AS in cells (Muraki et al. 2004). KH-CB19 also exhibits Clk inhibitory activity in cells. The selectivity profile showed strong inhibition of Clk4, which was evaluated by thermal displacement analysis of 106 kinases (Fedorov et al. 2011). Our study showed that CLASRP-overexpressing CRC cells were more sensitive to the inhibitor TG003 than to the inhibitor KH-CB19. The growth of CLASRP-overexpressing CRC cells stagnated at high concentrations of the inhibitor TG003 rather than those of the inhibitor KH-CB19, indicating that the inhibitor TG003 effectively inhibited the growth of CRC cells with CLASRP overexpression. The inhibitor TG003 can effectively downregulate the expression of CLASRP and control the growth of CLASRP-overexpressing CRC cells. Thus, CLASRP is a new target for the treatment of CRC.

We also noticed that high concentrations of Clk inhibitors could downregulate CLASRP expression in vector DLD-1 cells in terms of both gene and protein expression pattern modifications. However, low concentrations of Clk inhibitors significantly downregulated CLASRP expression in CLASRP-overexpressing DLD-1 cells. This means that the higher the CLASRP expression level is, the more sensitive it is to inhibitors. Compared to vector cells, in CLASRP-overexpressing DLD-1 cells, high expression of CLASRP serves as an efficient target for Clk inhibitors, which leads to a powerful inhibitory effect of Clk inhibitors on CLASRP. However, there may be an indirect interaction between CLASRP and Clk inhibitors because overexpression of CLASRP leads to changes in upstream and downstream pathways. Therefore, further research should analyse splicing to confirm a direct interaction between Clk(s) and CLASRP, which is an effective target inhibited by Clk inhibitors. Similarly, inhibition of Clk1 using TG003 and Clk1 siRNA resulted in a decreased cell viability, proliferation, invasion and migration as well as modulation in the phosphorylation of SRSF2, which validated the use of Clk1 as a potential target for gastric cancer treatment (Babu et al. 2020). Nevertheless, the inhibitor TG003 effectively controlled the growth of tumours and suppressed the expression of CLASRP in vivo, suggesting that CLASRP is a promotional oncogene that could be regulated by Clk inhibitors.

There are some limitations in this study. When studying the effects of Clk inhibitors on the biological functions of CLASRP, vector DLD-1 cells and CLASRP-overexpressing DLD-1 cells were used. It is not well explained why Clk inhibitors show a low effect on vector DLD-1cells, in terms of both cell proliferation in vitro and gene expression pattern modifications. This may actually suggest possible off-target and/or indirect effects, operated by the genetic modifications and/or by Clk inhibitors.

In summary, CLASRP is an oncogene that promotes the migration, invasion and proliferation of CRC cells. Our findings provide new insights into CLASRP as a novel therapeutic target that is effectively inhibited by Clk inhibitors when CLASRP is overexpressed in CRC.

## Supplementary information

ESM 1

## Data Availability

All datasets used and/or analysed during the study are available from the corresponding author upon reasonable request.
