# Supplementary material for: CLASRP oncogene as a novel target for colorectal cancer
Source: Funct Integr Genomics. 2023 Sep 2;23(3):290. doi: 10.1007/s10142-023-01208-8 (PMC10474993; doi:10.1007/s10142-023-01208-8)
Supplement: Supplementary file 1 — ESM 1 [file 10142_2023_1208_MOESM1_ESM.docx]

Table S1 Tumor score in xenograft with *CLASRP*-overexpressed DLD1 cells

| Groups | Morphological changes | | | Necrosis score |
| --- | --- | --- | --- | --- |
|  | Necrosis | Inflammatory cell infiltration | Apoptosis |  |
| Vector-1 | +++ | - | + | 3 |
| Vector-2 | +++ | - | + | 3 |
| Vector-3 | +++ | - | + | 3 |
| CMV-1 | ++++ | - | + | 4 |
| CMV-2 | ++++ | - | + | 4 |
| CMV-3 | ++++ | - | + | 4 |

Tumor score: The morphology of tumor cells, the degree of necrosis, interstitial blood vessels, hemorrhage and inflammatory cell infiltration were examined by light microscope. The degree of tumor necrosis was rated as 0-4 grade (score), and the proportion of tumor necrosis in tumor tissue was less than 1/4, which is evaluates as 1 point (1+, mild or small). About 1/4~2/4 of the necrotic tissue is 2 points (2+, moderate or more); About 2/4~3/4 of the necrotic tissue is 3 points +(3+, severe or excessive); > 3/4 is 4 points (4+, extremely severe or large).

Vecter, mice inoculated with vector DLD1 cells. CMV, mice inoculated with *CLASRP*-overexpressed DLD1 cells.

Table S2 The primer sequences

| Name | Primer | Sequence (5’-3’) |
| --- | --- | --- |
| Homo *GAPDH* | Forward | GGATTTGGTCGTATTGGGCG |
|  | Reverse | ATCGCCCCACTTGATTTTGG |
| Homo *CLASRP* | Forward | GACTACACCCCCCCTCTGCT |
|  | Reverse | CTCCTCCTCTGGCTTTTCTGC |

Table S3 The shRNA sequences

| Gene |  | Sequence |  |
| --- | --- | --- | --- |
| *CLASRP* | sh-1 | GCGAAGAUGAGAAGAAGAA | UUCUUCUUCUCAUCUUCGC |
|  | sh-2 | CGGACGAACGGAAGUGUAA | UUACACUUCCGUUCGUCCG |
|  | sh-3 | GCAUGAUGGUCGACUACAA | UUGUAGUCGACCAUCAUGC |


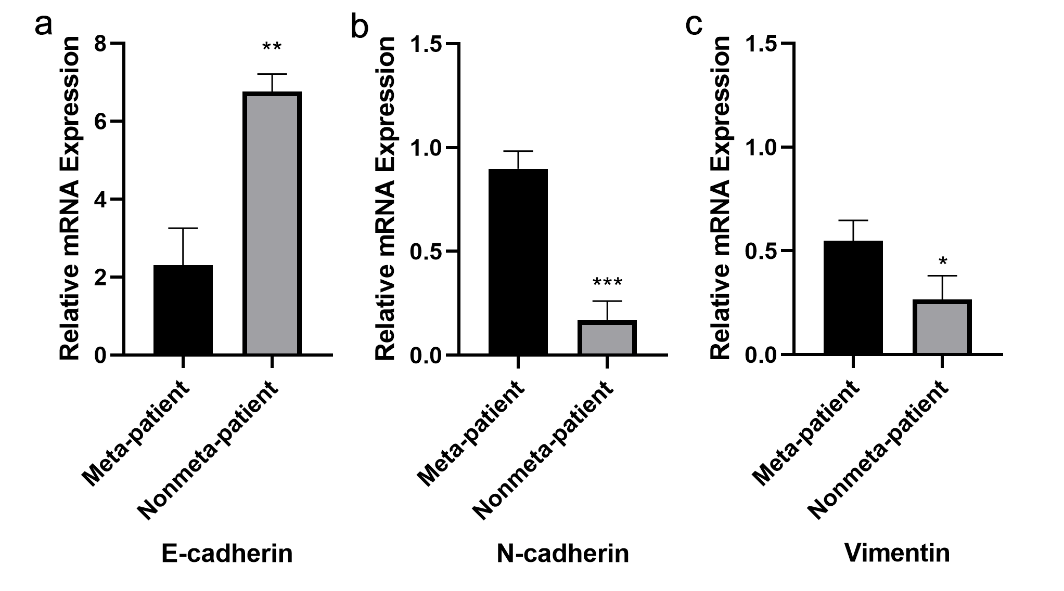


**Figure S1**: Analysis of EMT marker expression in CRC tissues. The relative mRNA expression of a) E-cadherin and b) N-cadherin and c) Vimentin in paired adjacent tissues from non-metastatic and metastatic patients. Meta-patient, patients with metastasis. Nonmeta-patient, patients with non-metastasis. Data are mean ± SD, n = 3. ***p < 0.001, **p < 0.01, *p < 0.05.


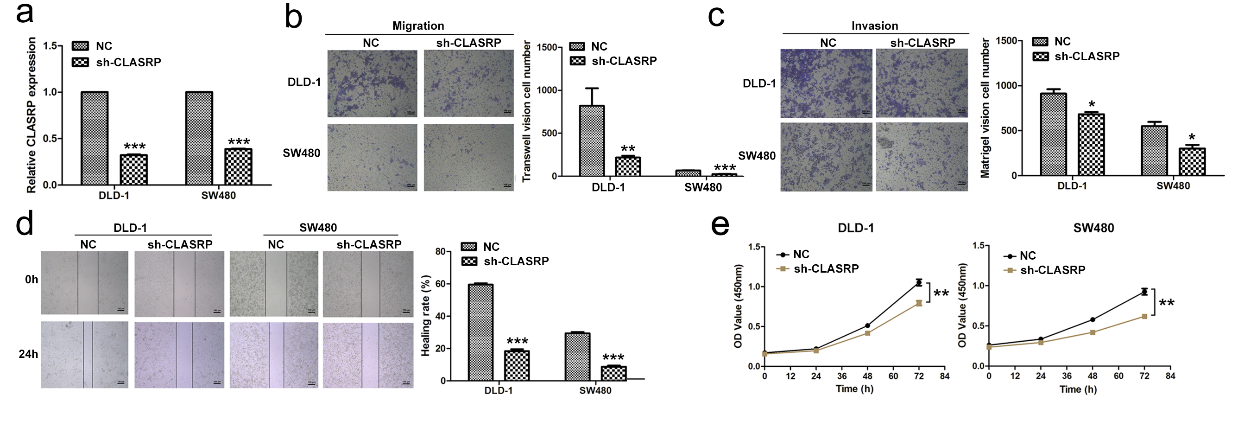


**Figure S2.** CRC cells transfected with *CLASRP* shRNAs. Interference of *CLASRP* suppressed migration and invasion in CRC cells with transfection of the shRNAs. a, the expression level of *CLASRP* in DLD-1 and SW480 cells after knocking-down by sh-*CLASRP*. b-e, the effect of *CLASRP* on cell migration and invasion, and proliferation ability were assessed by transwell migration, matrigel invasion assays, wound healing, and CCK-8 assay in *CLASRP* knocking-down DLD-1 and SW480 cells. Data are mean ± SD, n = 3. ***p < 0.001, **p < 0.01, *p < 0.05.


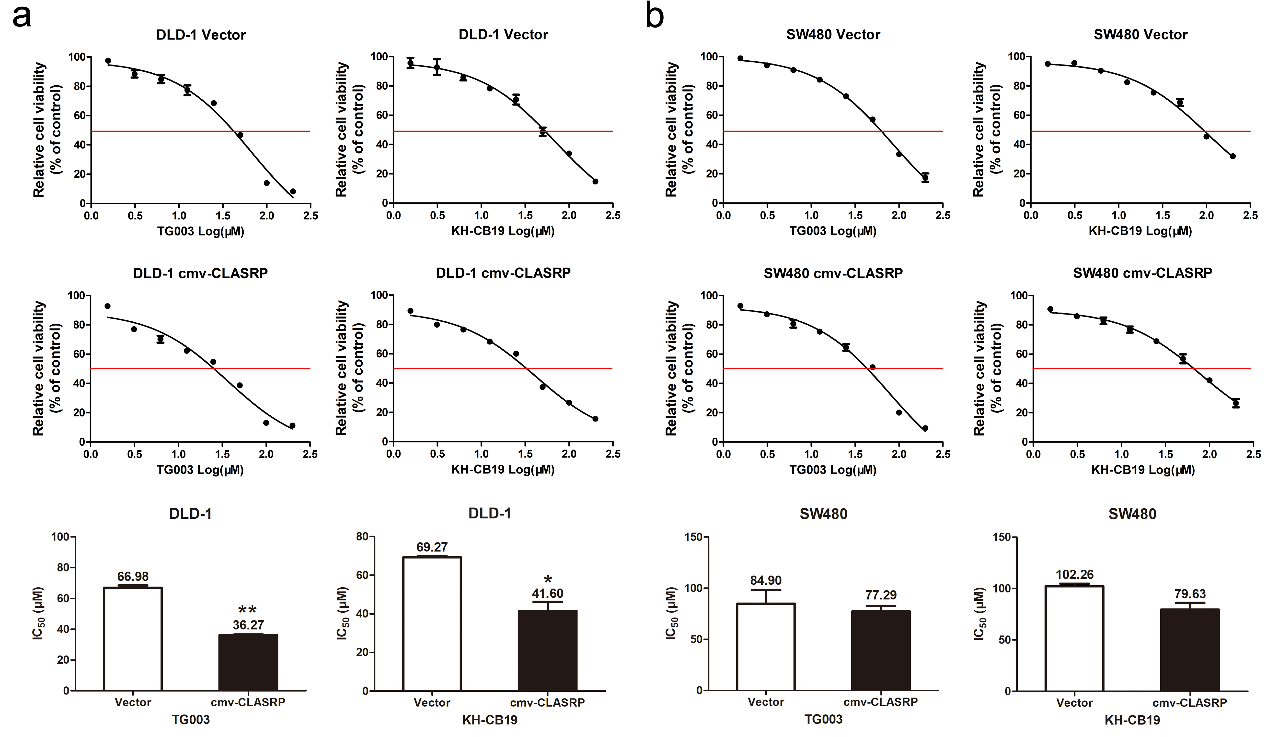


**Figure S3.** IC_50_ of Clk inhibitors to *CLASRP*-overexpressed CRC cells. IC_50_ of Clk inhibitors to vector and *CLASRP*-overexpressed a) DLD-1 and b) SW480 cells, respectively. Data are mean ± SD, n = 3. **p < 0.01, *p < 0.05.


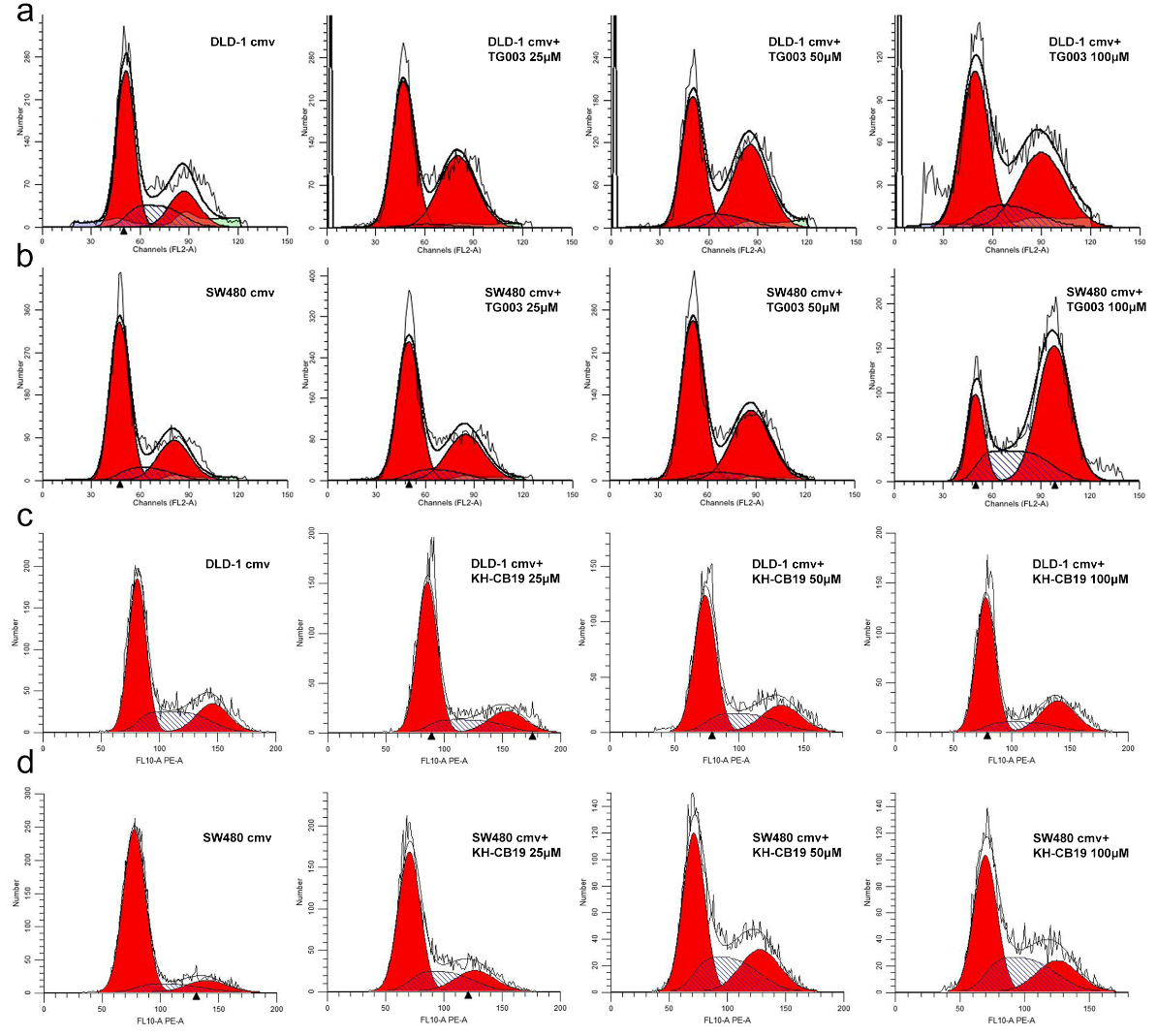


**Figure S4.** Flow cytometry of cell cycle phase distribution in *CLASRP*-overexpressed CRC cells treated with Clk inhibitors. a) *CLASRP*-overexpressed DLD-1 cells and b) *CLASRP*-overexpressed SW480 cells treated with inhibitor TG003. c) *CLASRP*-overexpressed DLD-1 cells and d) *CLASRP*-overexpressed SW480 cells treated with inhibitor KH-CB19. The original picture comes from one of three independent experiments. cmv, *CLASRP*-overexpressed DLD-1 cells without treatment.


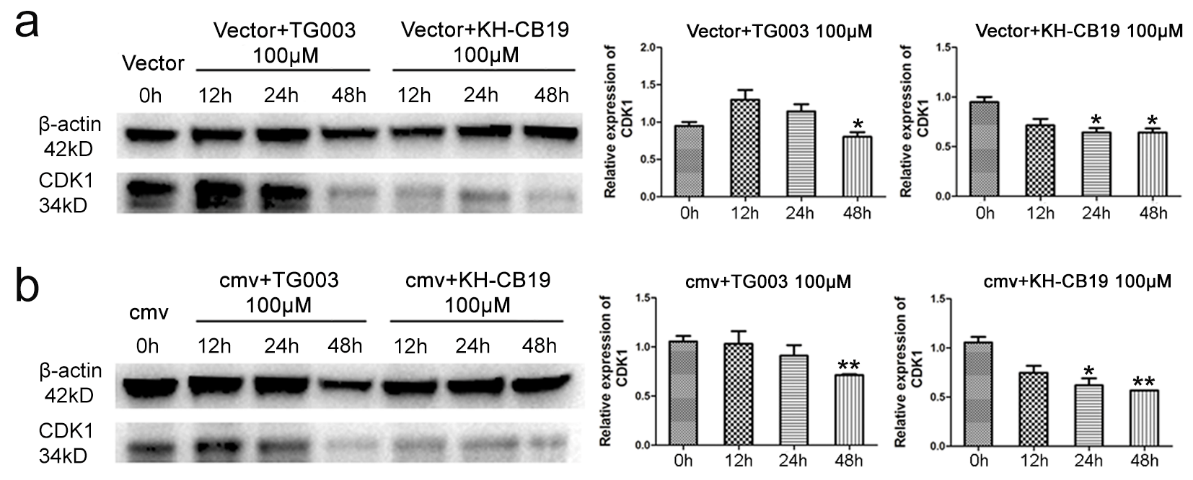


**Figure S5.** Clk inhibitors arrested the cell cycles in *CLASRP*-overexpressed CRC cells. The expression of CDK1 in *CLASRP*-overexpressed CRC cells treated with a) inhibitor TG003 and b) inhibitor KH-CB19 for 0h, 12h, 24h, 48h. cmv, *CLASRP*-overexpressed DLD-1 cells without treatment. Data are mean ± SD. n =3. ***p < 0.001, **p < 0.01, *p < 0.05.


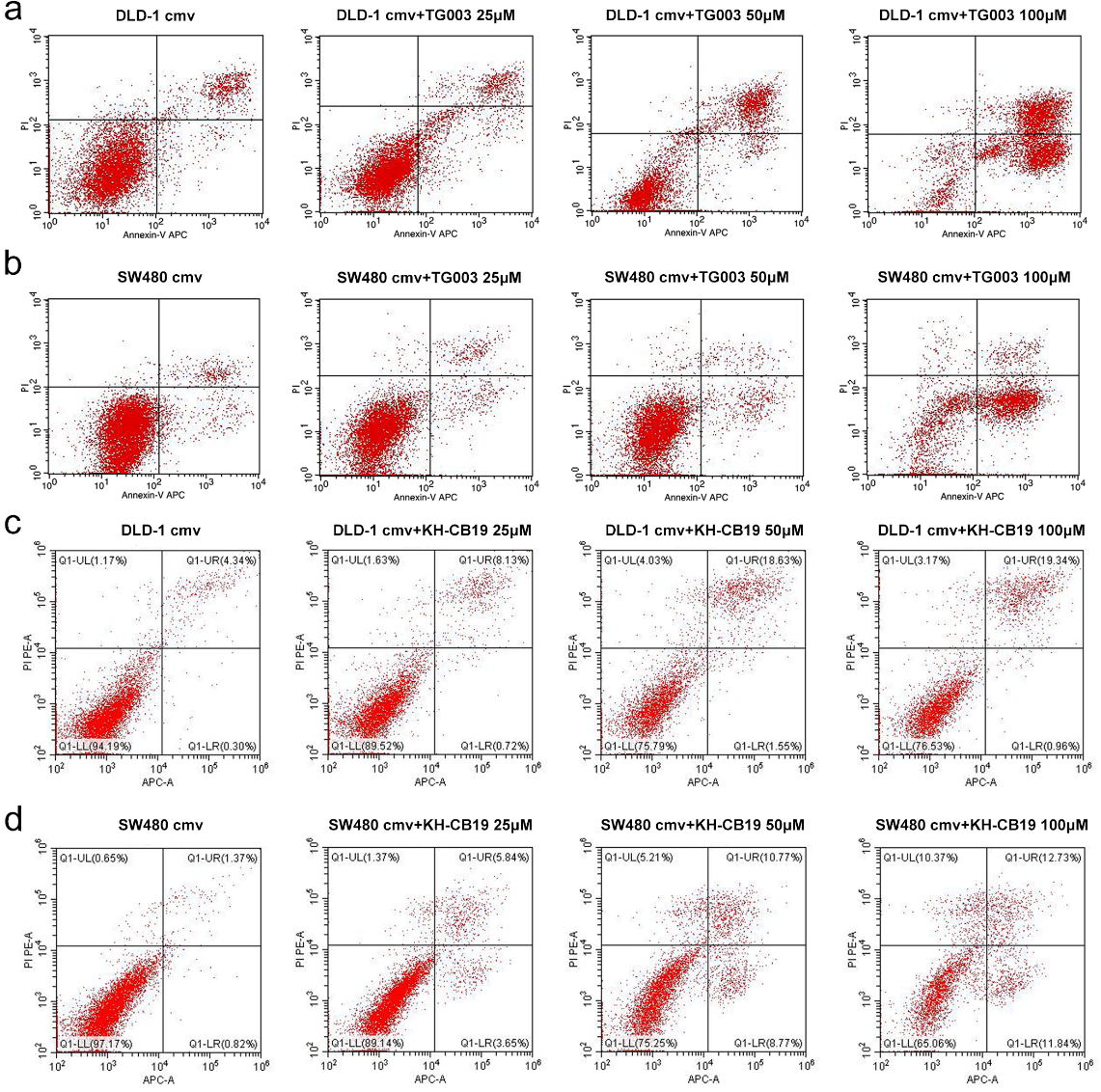


**Figure S6.** Flow cytometry of apoptosis in *CLASRP*-overexpressed CRC cells treated with Clk inhibitors. a) *CLASRP*-overexpressed DLD-1 cells and b) *CLASRP*-overexpressed SW480 cells treated with inhibitor TG003. c) *CLASRP*-overexpressed DLD-1 cells and d) *CLASRP*-overexpressed SW480 cells treated with inhibitor KH-CB19. The original picture comes from one of three independent experiments. cmv, *CLASRP*-overexpressed DLD-1 cells without treatment.
